# How the Use of a Patient-Accessible Health Record Contributes to Patient-Centered Care: Scoping Review

**DOI:** 10.2196/17655

**Published:** 2021-01-11

**Authors:** Janine Benjamins, Annemien Haveman-Nies, Marian Gunnink, Annemieke Goudkuil, Emely de Vet

**Affiliations:** 1 Icare JGZ Meppel Netherlands; 2 Chairgroup Consumption & Healthy Lifestyles Wageningen University Wageningen Netherlands; 3 GGD NOG Warnsveld Netherlands

**Keywords:** personal health records, patient portals, patient-centered care, patient-accessible records

## Abstract

**Background:**

Worldwide, patient-centered care is becoming a widely used concept in medical practice, getting more and more attention because of its proven ability to improve quality of care and reduce costs. Although several studies show that patient-accessible electronic health records (PAEHRs) influence certain aspects of patient-centered care, the possible contribution of PAEHR implementation to patient-centered care as a comprehensive concept has not, to our knowledge, been structurally evaluated to date.

**Objective:**

The objective of this study is to review whether and how the use of PAEHRs contributes to patient-centered care both in general and among specific population groups.

**Methods:**

We followed PRISMA Extension for Scoping Reviews reporting guidelines. We identified literature in 5 databases, using the terms “patient-accessible medical records,” “patient experiences,” and “professional experiences” as key concepts. A total of 49 articles were included and analyzed with a charting code list containing 10 elements of patient-centered care.

**Results:**

Studies were diverse in design, country of origin, functionalities of the investigated PAEHR, and target population. Participants in all studies were adults. Most studies reported positive influence of PAEHR use on patient-centered care; patient accessible health records were appreciated for their opportunity to empower patients, inform patients about their health, and involve patients in their own care. There were mixed results for the extent to which PAEHRs affected the relation between patients and clinicians. Professionals and patients in mental health care held opposing views concerning the impact of transparency, where professionals appeared more worried about potential negative impact of PAEHRs on the patient-clinician relationship. Their worries seemed to be influenced by a reluctant attitude toward patient-centered care. Disadvantaged groups appeared to have less access to and make less use of patient-accessible records than the average population but experienced more benefits than the average population when they actually used PAEHRs.

**Conclusions:**

The review indicates that PAEHRs bear the potential to positively contribute to patient-centered care. However, concerns from professionals about the impact of transparency on the patient-clinician relationship as well as the importance of a patient-centered attitude need to be addressed. Potentially significant benefits for disadvantaged groups will be achieved only through easily accessible and user-friendly PAEHRs.

## Introduction

In the last 30 years, patient-centeredness has grown worldwide in relevance in health care policy, practice, and research. In 1987, Harvey Picker developed the Pickers’ Principles of Patient Centered Care [1]. Thereafter, patient-centered care gained increasing prominence in the US when the Institute of Medicine advocated for patient-centered care as a cornerstone of health care quality [2]. In 2015, the World Health Organization stated that patient-centered care should become the standard for health care systems all over the world [3].

Key factors in patient-centered care are responsiveness to the patients’ individual needs and preferences, and partnership between care providers and patients in decision making [4-7]. Patients are acknowledged as unique human beings with needs and preferences that have to be taken into account when clinical decisions are made. Ideally, patients as well as their family members or caregivers are involved in making these decisions. This requires clear information and communication with patients.

Patient-centered care has been gaining importance because of its proven ability to increase the quality of care, with lower health care utilization as a beneficial side effect [3,8-13]. The growing importance and development of the concept in different countries has led to a diversity in models, definitions, and terminology. For this review, we used an integrative model by Scholl et al [5], integrating more than 400 definitions and models into a new and comprehensible model for patient-centered care.

In the Netherlands, patient-centered care has also taken center stage in the discussion about quality of care, especially in care for youth [14]. To contribute to patient-centered care, three organizations for preventive youth health care and youth social services in the North Veluwe region developed a PAEHR system [14]. The assumption that the use of PAEHRs contributes to patient-centered care, however, has not yet been sufficiently proven.

Several reviewers investigated effects of PAEHRs by reporting on a variety of outcomes related to patient health, quality of care, or patient satisfaction [15-23]. The aspects of patient-centered care that have been mentioned are, for instance, empowerment of patients, trust in care providers, and the clinician-patient relationship. For these aspects, both beneficial [15-19] and unfavorable or even harmful consequences of the use of a PAEHR [19-23] to patient-centered care have been reported. Some studies report that disadvantaged groups might benefit less from the use of PAEHRs than others, as their access to and use of PAEHRs is lower than average [19,20,22,23]. To date, we know of no published review that structurally evaluates the possible contribution of PAEHRs to patient-centered care as a comprehensive concept. Performing such a review would enable us to explore whether PAEHRs could serve as a tool to strengthen this value-based health care model.

Since the relationship between the use of PAEHRs and the broad concept of patient-centered care has, to date, received limited attention in reviews, a broad overview of recent literature is required, with inclusion of different study designs. With such a broad perspective, a scoping review is more suitable than a systematic review, as scoping reviews aim to broadly summarize and synthesize evidence instead of finding answers to circumscript questions and including only specified study designs. A scoping review can be helpful to provide direction to future research and search for gaps in knowledge [24,25]. The objective of this review is to provide an overview of recent literature about experiences of patients and professionals with the use of PAEHRs and to investigate whether and how the use of PAEHRs contributes to patient-centered care, both in general and among specific population groups.

## Methods

### Search Strategy and Inclusion Criteria

Design and reporting of this scoping review were in line with the framework for scoping reviews by Arksey and O’Malley [24-26], which was further developed by other authors, finally leading to the PRISMA Extension for Scoping Reviews guideline and checklist [27,28]. Multimedia Appendix 1 contains the completed PRISMA checklist for this review. The a priori review protocol has not been registered. Key concepts used in the search were “patient-accessible medical records,” “patient experiences,” and “professional experiences.” Table 1 contains the full electronic search string for the Scopus database. The search was limited to papers written in English or Dutch, being languages all authors understand, and to studies published between January 2000 and April 2019. This period was chosen because, in a first quick search, most articles about PAEHRs appeared to originate from 2000 or more recently. Five databases were searched: (1) Pubmed, (2) Medline, (3) Scopus, (4) Socindex, and (5) Psychinfo. The final search was run on April 9, 2019. Search records were uploaded to Endnote X8 to facilitate the article selection process.

**Figure 1:**
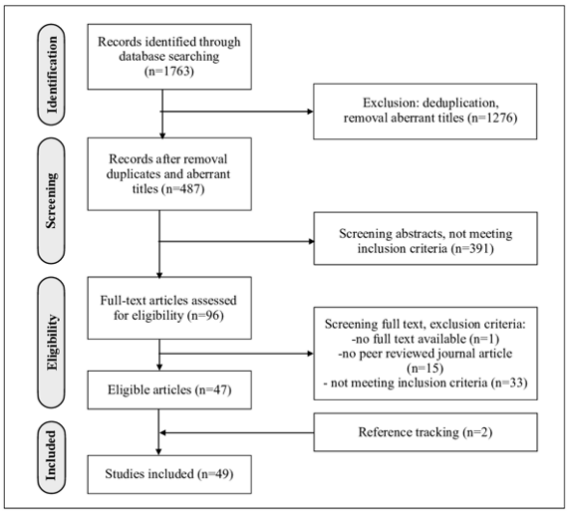
Flow diagram of article selection.

Searches, deduplication, and first screening of titles were performed by SJB. In total, 1763 articles were found and screened for eligibility (Figure 1). Aberrant titles were removed, and abstracts of remaining articles were independently screened by different individuals (SJB, MG, and AG), in line with the scoping nature of the review. We included research articles from peer reviewed journals for which full text could be retrieved. The articles were based on original research data. They addressed “experiences” of professionals or patients/clients using a PAEHR. Articles were screened in 3 rounds. After every round, different interpretations were discussed between all three screening authors to come to a unanimous decision. If necessary, the inclusion criteria were adapted before the next round to ensure uniform selection. SJB screened the remaining full text articles on inclusion criteria. To exclude articles from predatory journals, every journal was checked against the JournalGuide whitelist [29]. The selection process was finalized by reference tracking; all references of selected articles were checked with the inclusion criteria and added when eligible.

**Table 1:** Full search string for Scopus, split into three key concepts.

Key concepts	Search string per concept
Patient-accessible	(“Patient” OR “Patients” OR “client” OR “clients”) AND (“access” OR “online access” OR “accessible”) AND (“record” OR “records” OR “file” OR “files”)
Medical records	AND “Personal health records” OR “Health Record, Personal” OR “Personal Health Record” OR “Record, personal health” OR “personal health records” OR “Personal Health information” OR “Health Information, Personal” OR “Information, Personal Health” OR “Personal Medical Records” OR “Medical Record, Personal” OR “Medical Records, Personal” OR “Personal Medical Record” OR “Record, Personal Medical” OR “Records, Personal Medical” OR “patient portals” OR “Patient Web Portal” OR “Portal, Patient Web” OR “Portals, Patient Web” OR “Web Portal, Patient” OR “Web Portals, Patient” OR “Patient Internet Portals” OR “Internet Portal, Patient” OR “Internet Portals, Patient” OR “Patient Internet Portal” OR “Portal, Patient Internet” OR “Portals, Patient Internet” OR “Patient Web Portals” OR “Patient Portal” OR “Portal, Patient” OR “Open Notes” OR “Electronic health records”
Patient experiences AND physician experiences	AND “patient experiences” OR “physician experiences” OR “experiences” OR “experiences, patient” OR “experiences, patients” OR “experiences, physician” OR “experiences, physicians” OR “experiences, professional” OR “professional experiences” OR “outcome assessment (health care)” OR “benefit” OR “satisfaction” OR “patient outcomes”

### Data Analysis

**Figure 2:**
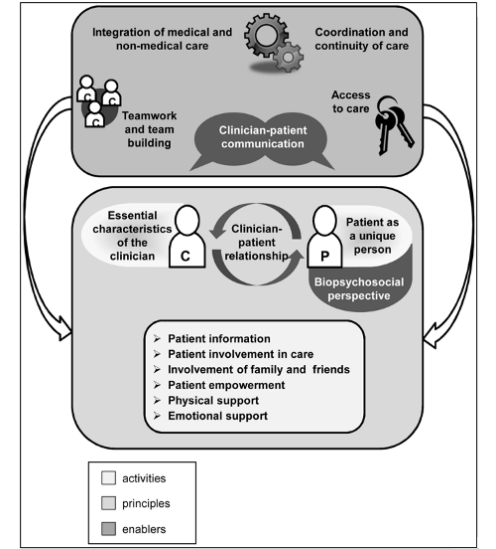
Model of Patient-centered Care, by Scholl et al (2014).

Through discussion SJB, AH, and EV came to a charting code list (see Multimedia Appendix 2). The list contained codes for general article information, study methods, description of the investigated PAEHR, and 10 dimensions of patient-centered care. The dimensions of patient-centered care were derived from a model, developed by Scholl et al (Figure 2) [5]. This model distinguishes 15 dimensions in 3 groups: (1) principles, (2) enablers, and (3) activities. The principles represent the essential factors of a patient-centered attitude in professionals. The principles and the enablers, which are organizational conditions for patient-centeredness, lay the foundation for the last group, the activities. These are actions and measures by which patient-centered behavior becomes visible. Assuming that use of PAEHRs would affect the “activities” from the model, possibly affect the “enablers,” and not affect the “principles,” we included all 5 enablers and 4 activities. We did not include the activities “physical support” and “emotional support,” since we expected not to find any relation with the use of PAEHRs. From the principles, only clinician-patient relationship was included, because we considered this dimension a dynamic one that could be influenced by use of a PAEHR. A separate charting code was created for differences among population groups, since former research suggests that disadvantaged groups might benefit less from the use of PAEHRs than others [19,20,22,23]. The charting process was done by SJB and discussed afterward with the other authors. All charted data were aggregated through group discussion with all co-authors.

## Results

### Overview

In total, 49 eligible articles were included [21,30-77]. Multimedia Appendix 3 presents a brief summary of the articles, with characteristics of each study, functionalities of the studied PAEHR, and reported elements of patient-centered care. Multimedia Appendix 4 provides an overview of all outcomes. In this appendix, the articles were divided into 3 study design groups to facilitate the analysis. The largest group (n=34) consists of descriptive studies, both qualitative and quantitative [21,32-35,37-43,45,47-51,53,55,56,58,59,62,63,65,69-73, 75-77]. The other 2 groups contain pre-post-test comparative studies [21,40,60,61, 70,71,75,76] and studies comparing intervention and control groups [30,31,36,44,46,47,52, 54,57,64,66-68,74]. The results of 7 mixed methods studies were divided and categorized according to the groups they best matched with [21,40,47,70,71,75,76].

Most articles (n=29) originated from the US [21,30,32-40,42,44-46,49,50,54-58,60,63,66,69,74,76,77]. Clustered in 5-year periods, 3 articles originated from 2000-2004 [34,40,66], 3 from 2005-2009 [43,67,68], 15 from 2010-2014 [21,33,47,49,54,55,58,59,64,69,71,73-75,77], and 28 from 2014-2019 [30-32,35-39,41,42,44-46,48,50-53,56,57,60-63, 65,70,72,76]. Duration of experience with a PAEHR varied from 1.5 to 48 months. Population sizes were also diverse, ranging from 9 in a qualitative study [41] to several thousand in an Open Notes survey study (n=29,191) [56]. Finally, the population demographics varied; most studies included a broad range of patients (eg, patients in hospitals [30,34,60,72,76] or in primary care [21,32,42-46,48,49,54,57,58,63]). Other studies focused on specific patient groups, like cancer patients [30,37,50,59,62,75], cardiac patients [40,66,74], chronically ill patients [31,64,71], HIV-positive patients [36,57], psychiatric patients [35,39,70], gynecologic patients [67,68,73], and veterans [35,36,39,55,56,69,77]. Ten studies investigated experiences of both patients and their care providers [21,32,34,40,51,57,58,60,70,72]. Four studies focused on professionals only [38,41,61,65]. Respondents in all studies were adults, mostly of no specific age group. Three studies surveyed parents of pediatric patients [33,37,51].

Apart from record-access, the most common functionalities of the PAEHRs were “electronic messaging” [33,36,37, 40,51,55,56,64-66,68-70,72,74,76] and the possibility to add or edit health information [31,34,54-56,58,62,65,70,72,74,75]. Six studies investigated a so-called active PAEHR that sent patients “personalized health messages” [31,34,58,63,67,68]. Other functionalities were “give feedback on health information”[62,65], “download information to share with others”[30,42,58], “grant direct access to others” [55,62,76,77], and administrative tasks like “scheduling appointments” [30,51,59], “paying bills” [30], and “requesting medication refill” [30,72].

One patient-accessible record was paper-based and consisted of a briefcase with all medical information, which was updated after every visit to the clinic [47]. Two PAEHRs were electronic but not available online [43,73]. One was a USB-stick containing all medical information, which was revised during every visit to the clinic [73]. The other was a kiosk in the clinic’s waiting room, where patients could access all medical info during their visit [43]. In one study, 9 physicians were interviewed about their experiences with PAEHRs in general [30].

### Dimensions of Patient Centeredness

The outcomes for the 10 coded dimensions of patient-centered care have been summarized in Table 2. In 34 of the studies at least 3 of these dimensions were explored. None of the studies mentioned the dimensions “integration of medical and non-medical care” and “teamwork”. The following paragraphs describe the outcomes for each dimension of patient-centered care. When describing outcomes, we use the term “effect” both for experienced effects as well as for objective results from comparative studies.

**Table 2:** Summary of results for dimensions of patient-centered care. This table represents, for every explored dimension of patient-centered care, whether reported outcomes point in a positive or negative direction. “Negative” in a pre-post comparative design means “less positive than expected.” In a pre-post or intervention-control design, the term “neutral” refers to the outcome “no difference” or “no significant difference.”

Dimension	Number of studies, n	Descriptive studies, reference number		Comparative studies, reference number		
		Positive	Negative	Positive	Neutral	Negative
Information	40	[21,32-34,37-43,45,47-51,53,55,56,58, 59,62,63,65,69-72,75-77]	N/A^a^	[30,31,36]^b^; [44,46,47,76]	[60,67]	[61]
Involvement in care	33	[32,34,37,38,40-43,47-51,55,56,59,62, 63,65,69,71,75,77]	N/A	[36,54]^b^; [44]	[30,46,64,67,74]	[60,61]
Empowerment	23	[21,33,39,42,45,47,48,50,56,58,63,70,76]	N/A	[46,59,60,66]; [76]^b^	[36,40,61,68, 70,71,75]	N/A
Communication	22	[33,34,37,40,41,45,47,48,51,53,55,58,59, 62,63,65,70,71,76,77]	N/A	[76]	[57,66]	N/A
Involvement of family and friends	14	[42,45,47,49,51,55,59,62,69,71,73,76]; [70]^c^	[70]^c^	[57]	N/A	N/A
Clinician-patient relationship	22	[21,32,35,38-43,45,50,59,62,63,65,71,72]	[41]^c^	[44,74]	[57]	[60,61]
Access to care	5	[42,45,49,62,63]	N/A	N/A	N/A	N/A
Coordination / continuity of care	3	[40,58,76]	N/A	N/A	N/A	N/A
Integration medical / nonmedical	0	N/A	N/A	N/A	N/A	N/A
Teamwork	0	N/A	N/A	N/A	N/A	N/A

^a^N/A: not applicable.

^b^Significant effect.

^c^Both positive and negative aspects reported.

#### Information

Forty studies investigated if and in what way patients felt more informed about their health after use of a PAEHR. We distinguished 3 different topics: (1) what patients valued in reading records, (2) emotional consequences, and (3) understandability. Seven descriptive studies examined reasons for reading medical records [32,43,45,56,62,75,76]. Patients valued reading their record because they wanted to know about their health or because they wanted to be sure they understood what the doctor said or because they were curious. Patients valued reading their records most because it improved understanding of health issues [21,34,39, 45-47,50,53,56,60,61,65,69,71,75-77], helped to prepare for next visits [21,56,59,61-63,65,71,75,76], and helped to remember the care plan [21,40,42,43,45,46,49,50,56,61,76]. Reading also helped patients to follow treatment recommendations [33,39,41]. Six studies compared the difference in health knowledge between intervention and control groups [30,31,36,44,47,67]. One study found a significantly higher “self-health management knowledge score” among PAEHR adopters than among nonadopters (*P*<.01) [30]. Another study found that the intervention group was significantly better informed than the control group about their latest blood measurement levels, including date, time, and trend changes, and about normal lab values (*P*<.001) [31]. A third study found that HealtheVet users were able to correctly identify their CD4 counts significantly more often (Fisher exact test=.048) and their viral load (Fisher exact test=.003) than nonusers [36]. The other studies found no significant difference [44,47,67]. Two pre-post studies compared expectations with experiences [61,76]. After a period of PAEHR use, one of the studies reported better understanding of care plans among patients than expected (OR=1.39) [76]. In the other study, however, interviewed psychiatrists reported less improvement than expected in the extent to which patients understood their medical conditions or remembered their care plans [61].

Reading their records also provided patients with reassurance [33]. In 4 qualitative studies, patients said that transparency reduced anxiety and stress [33,45,56,62]. They experienced waiting for news as more stressful than reading notes by themselves. One patient said: “It is easier to break down at home where you are surrounded by family, than at the doctor’s office” [62]. If reading records caused stress, this was in most cases related to new diagnoses which had not yet been discussed with the professional [33]. Stress was also caused if health care professionals trivialized a patient’s problem in the record [39]. Less than 10% of patients often or always experienced worries or confusion after reading their record [21,39,56,57,76]. Three intervention-control studies found no significant difference in anxiety levels or reported worries between users and nonusers [47,52,57].

Six studies investigated if patients understood everything they read and how they felt they did not understand [34,40,50,58,62,77]. Some patients said they would appreciate built-in-definitions and less jargon. On the other hand, one patient added: “I would rather have the doctors just write what they write and me work to understand it, than them writing it for me and leaving something out that I would like to know” [40]. Moreover, although patients found some medical terminology too difficult, they managed to find explanations on the internet [58,62].

#### Involvement in Care

Thirty-two studies described the impact of use of PAEHRs on involvement in care. Twenty-three descriptive studies described involvement of patients in their care as a benefit of using a PAEHRs [32,34,37,38,40-43,47-51,55,56,59,62,63,65,69,71, 75,77]. Clinicians in one study said that using a PAEHR resulted in a “power shift” towards patients. Some of them saw this as a “move towards patient-centered care, creating better opportunities for collaboration with patients” [38]. In intervention-control studies, the 13-question Patient Activation Measurement (PAM-13) Questionnaire was most commonly used to measure involvement of patients in their care. Two intervention-control studies found a significantly higher PAM-score in the user groups [36,54]. One study reported a mean PAM-13 score of 47 points in the intervention group versus 45 points in the control group (*P*=.0014) [54], whereas the other study reported a mean PAM-13 score of 72.5 in the intervention group versus a mean of 63.49 in the control group (*P*=.03) [36]. Three studies found no significant effect on activation score or decision making [64,67,74]. One study, comparing different user subgroups, reported that less educated patients and non-White patients were more likely to report that reading visit notes was extremely important to engaging in their care than more educated and White patients [46]. In the 2 pre-post comparisons, the observation that patients were “feeling more in control” was slightly lower than expected [60,61].

Five studies investigated if patient involvement would result in patients finding and correcting errors in their record [45,60,62,65,76]. One descriptive study reported that 6 patients in a group of 15 had found errors but had not requested correction [62]. One study investigated a PAEHR with a feedback option [45]. Patients valued this feedback option because it helped them to correct errors. Two descriptive studies reported that physicians felt that use of PAEHRs could prevent medical errors and that the PAEHRs were used by patients as a means to check for accuracy [65,76]. In one pre-post study, patients found less errors than expected, although errors were found and corrected; in a group of 50 patients, 3 patients reported finding errors in medication, 2 patients found errors in radiology test reports, and 1 patient found an error in a laboratory test report [60].

#### Involvement of Family and Friends

Fourteen studies investigated whether and how family and friends were involved in care through use of PAEHRs. Thirteen descriptive studies reported that patients shared health information with relatives, friends, and health professionals [42,45,47,49,51,55,59,62,69-71,73,76]. Patients said they shared information to answer questions of family and friends and to keep them informed. Sharing information also helped to discuss their disease with relatives or caregivers. The percentage of patients who actually shared notes with others differed among studies, from 15% to 67%. One descriptive study among patients with a bipolar disorder reported that 23% of the 39 respondents considered access to family caregivers preferable, whereas 25% thought it would be harmful [70]. One study, comparing HIV-positive patients with other patients in primary care, found that HIV-positive patients were more likely than other primary care patients to share or discuss visit notes with others, both friends and professionals [57]. In one mixed-methods study, caregivers especially valued the ability for a patient to share information with them, because this enabled them to view notes of visits which they had not been able to attend [76].

#### Empowerment

In 13 descriptive studies, patients mentioned that they felt more in control of their health or that they could take better care of their own health due to reading their record [21,33,39,42,45,47,48,50,56,58,63,70,76]. In one study, patients appreciated the possibility to share a print-out of their record with another doctor [59]. Patients also said that their role became more active [45]. They experienced more ownership of their own health status [63]. Three control-intervention studies reported no significant difference in empowerment between intervention and control groups [36,66,68]. In 7 pre-post studies, 6 studies found no significant effect on empowerment scores [40,60,61,70,71,75]. The 7th study reported that patients were more confident in their ability to manage their health information (OR 2.14, 95% CI 1.59-2.89) and their care (OR 1.48, 95% CI 1.14-1.93) [76].

#### Communication

Twenty descriptive studies investigated the effect on communication between patient and health care professional and reported an improvement [33,34,37,40,41,45,47,48, 51,53,55,58,59,62,63,65,70,71,76,77]. Communication became easier because of the PAEHRs, and interaction improved [34,58]. The ability to view health information improved the level of communication during subsequent visits and made it possible to communicate “on a more level playing field” with health care professionals [41,51]. The use of a PAEHR also removed barriers, for instance, “because you can ask ‘stupid’ questions that you wouldn’t pick up the phone for” [33]. Two intervention-control studies reported on communication and found no significant differences between intervention and control groups [57,66]. One pre-post study reported that caregivers appreciated the possibility to view notes of visits they could not attend, because it improved their communication with care providers [76].

Seven descriptive studies investigated the influence of PAEHR use on time investment, 5 of them reporting no difference [21,32,40,58,62,65,72]. One study reported that some professionals needed more time to edit or explain notes. However, they framed this as “better documentation, a good thing” [21]. In one study, a professional said that it was improving efficiency: “finally something to save me time!” [58]. One intervention-control study reported that professionals received more messages per patient, but nonetheless did not feel a perceptible change in workload [66]. Four pre-post studies investigated expectations of more time investment, but none demonstrated an increased time investment [21,40,60,61].

#### Clinician-Patient Relationship

Seventeen descriptive studies reported on the clinician-patient relationship [21,32,35,38-43,45,50,59,62,63,65,71,72]. Patients reported that they were feeling better about their doctors after reading their records [32,39]. They appreciated their doctors’ expertise more and experienced a more equal relationship [40,41,43,45,62,64,65,72]. They valued the level of transparency, especially when notes were written respectfully [35,43,50,59]. Respectfully written notes contributed to their feelings of trust [35,71]. As a result, they felt heard and cared for [45]. Three intervention-control studies and 1 pre-post study reported on the professional-client relationship and found no significant differences [44,57,74]. Two other pre-post studies, however, found that the experienced increase of trust in physicians was less than expected, both from a patient and a professional perspective [60,61].

Related to the fear of damaging a therapeutic relationship, some professionals expected that they would report differently if they knew patients could be reading their visit notes. A psychiatrist in one study said: “Sometimes a disbalance occurs, patients ‘directing their care’ and dictating their doctors how to write their notes” [41]. These psychiatrists also feared that transparency of records could damage the therapeutic relationship, especially when notes revealed subjective impressions. Four pre-post intervention studies investigated if clinicians reported differently about sensitive subjects. Professionals appeared to report less differently than they had expected [21,57,58,61].

#### Access to Care

An access to care dimension was mentioned in 5 qualitative studies [42,45,49,62,63]. Patients experienced that the PAEHRs gave easy and quick access to health information [42,45,62]. Rapid access was perceived to be advantageous in emergency situations [49]. One study also mentioned that immediacy of secure messaging cultivated a sense of ease of access [63].

#### Coordination and Continuity

In 2 qualitative studies [40,58] continuity and coordination of care came up. Patients mentioned the benefit of being able to bring their health information along to another care provider and to take care of their own medication when they are out of town.

#### Differences Among Population Groups

Since former research suggests that different population groups do not profit equally from the use of PAEHRs [19,20,22,23], we searched for differences in our review. Seven studies compared the composition of the studied population with national demographic data. They reported that PAEHR users were more likely to be White and higher educated than nonusers [30,35,36,39,40,44,45]. Four studies investigated experiences of different ethnic and socioeconomic groups [32,45,46,49]. One descriptive study found that women, older patients, and high frequency users found reading notes very important to engaging in their care [45]. Another descriptive study reported that older, lower educated, retired, and unemployed patients, as well as patients with a poor self-reported health and participants in other studies were more willing to share visit notes with others [49]. A third descriptive study found that disadvantaged groups such as the elderly, non-White patients, less educated patients, or patients with poor self-reported health, reported more often than others that use of a PAEHR made them feel better about their doctors [32]. One intervention-control study focused on the importance of PAEHRs to non-White and less educated patients [46]. Both non-White and less educated patients reported more often than White and higher educated patients that the PAEHRs helped them to understand and remember care plans, feel informed, and make decisions concerning their own care. Both non-White patients and less educated patients found reading notes extremely important to engaging in their care.

## Discussion

### Summary

This review investigates whether and how the use of PAEHRs contributes to patient-centered care, both in general and among specific patient groups. Overall, the articles in this review support the assumption that patient-accessible records contribute to patient-centered care. In all 34 descriptive studies, a positive effect is reported for different dimensions. One descriptive study reported a possible negative effect of PAEHRs on the “therapeutic relationship.” Five out of 22 pre-post or intervention-control studies reported significant positive effects related to the dimensions “information,” “involvement of patients,” or “empowerment.” No significant negative effects were reported.

The studies in this review included adults only. Four studies found that, in particular, disadvantaged groups experienced PAEHR-related benefits [32,45,46,49].

### Dimensions of Patient-Centered Care

As we expected, the effect on the different “activities” in the Scholl et al model [5] was described most often. Although some effects on “enablers” are reported, only two of the “enablers” are mentioned: (1) access to care [42,45,49,62,63] and (2) coordination/continuity of care [40,58,76]. A complicating factor in the analysis was the varied use of dimensions and their definitions. For instance, whereas Scholl et al [5] distinguished “information,” “involvement in care,” and “empowerment” as different dimensions, some studies included “involvement” and “knowledge/information” in questionnaires about “empowerment” [5,40,68,71].

Furthermore, we found topics in our review that were not described by Scholl et al [5]. One topic was that patients contributed to patient safety by finding and correcting errors in their records [45,60,62,65,76]. After discussing this topic, we added the subject to “involvement in care,” arguing that patients showed their involvement in care by checking their record for errors. In a recent article by Zeh et al [78], however, patient safety was added to the Scholl et al model [5] as a new dimension based on a Delphi study among patients. Patients regarded patient safety as an important dimension of patient-centered care.

Both negative and positive effects were reported for the dimension “patient-clinician relationship.” In particular, professionals in mental health care expressed concerns that the transparency of PAEHRs would damage the patient-clinician relationship [38,61]. This is in line with results from other studies. In a recent Norwegian study [79], professionals in mental health care report significantly more often than their colleagues in somatic care that they change their way of writing when using PAEHRs. They also discuss significantly more often than their colleagues in somatic care whether patients should be denied access to their record. Dobscha et al [80] reported that only half of the mental health professionals they queried (107/198) considered sharing mental health Open Notes with patients a good idea, while most of them (174/205) supported the idea in general to share medical notes with patients.

In opposition to professionals, mental health care patients in our review felt that transparency in a PAEHR strengthened the patient-clinician relationship, given that sensitive information was reported in a respectful way [35,38]. The fact that professionals see this differently could be caused by traditional role expectations “in which the patient is viewed as someone to ‘protect’ and for whom the clinician is responsible” [38]. These role expectations are at odds with the patient-centered care principle of “equal partnership between client and professional” and might cause the reluctance toward the use of transparent PAEHRs.

In line with this assumption, another study emphasizes the importance of a patient-centered attitude by offering specific recommendations for mental health professionals to strengthen the therapeutic alliance in the context of patient-accessible records [35]. These recommendations focus on the “principle” dimensions from the Scholl et al model [5]. The findings in these studies strengthen the assumption in the Scholl et al model that the “activity” dimensions only become visible if the “principles” of patient-centered care, reflected in a patient-centered attitude, have been embraced by professionals.

### Differences Among Population Groups

Previous research suggests that disadvantaged groups might profit less from the introduction of PAEHRs than others because they make less use of PAEHRs [19,20,22,23]. In our review, 7 studies reported that users of PAEHRs were more likely to be White and higher educated than nonusers [30,35,36,39,40,44,45], probably due to different access abilities [36]. Surprisingly, 4 other studies found that disadvantaged groups experienced heightened benefits from the use of PAEHRs [32,45,46,49]. An explanation for this benefit could be the value of rereading information that cannot be absorbed all at once. Moreover, Bell et al [32] state that non-White patients are said to distrust White medical professionals, not expecting them to respect their cultural values. Reading transparent records would prove otherwise and might help these patients to trust their doctors more [32]. These findings show that disadvantaged groups benefit from the use of PAEHRs, once they have found their way into the system. This emphasizes the importance in designing and implementing PAEHRs that are easily accessible in order to include disadvantaged groups.

### Practical Implications

Our review shows that the use of PAEHRs could enhance patient-centered care, but the effects can be influenced by factors on professional and patient levels. On a professional level, adoption of the principles of patient centered care appears to be crucial for a positive impact of the use of PAEHRs on the patient-clinician relationship. On the patient level, easy access and user-friendliness is important to secure access for all demographics and to facilitate the PAEHR-related benefits that disadvantaged groups might experience.

### Strengths and Limitations

One of the strengths of this scoping review is that we included all types of designs and we did not focus on “patient-centered care-specific” search terms. As a result, we created a broad overview on the topic. Subsequently, the analysis was guided by the use of selected dimensions of patient-centered care from Scholl et al [5], which helped us to organize and interpret the information and added strength to the review. On the other hand, the fact that the analysis was conducted in separate dimensions made it more difficult to explore interaction and dependence between the dimensions and to draw conclusions about the impact of PAEHRs on patient-centered care as a whole.

Another strength is the combination of searches from 5 different databases, from both a medical and a social perspective.

A limitation of this review is that, by specifying only “physicians” in our search terms and not “nurses,” “nurse practitioners,” or nonmedical professionals, we could have missed some articles that were relevant to the subject.

One more limitation of this review is that we included articles in only English and Dutch and no unpublished data or grey literature. For example, no articles from Estonia or Japan could be included, although both countries are very active in eHealth and the government of Estonia has implemented a PAEHR system that is being used for every citizen of the country.

The strength of the conclusions in this review also depends on the quality of the individual studies. Therefore, we conducted a global quality check, where aspects of study design and population were assessed. Although a thorough quality appraisal is not common in scoping reviews, a more detailed quality check could have added strength to the review. The global check indicated that, on average, study results could have been biased because of population selection, as virtually all studies included only native speakers and most of the studies made use of convenience sampling.

### Conclusions

This review indicates that PAEHRs bear potential to positively contribute to patient-centered care. However, concerns from professionals about the impact of transparency on the patient-clinician relationship as well as the importance of a patient-centred attitude need to be addressed. Potentially high benefits for disadvantaged groups will be achieved only through easily accessible and user-friendly PAEHRs.

## Appendix

Multimedia Appendix 1 Completed PRISMA-ScR Checklist.

Multimedia Appendix 2 Coding list, used for analysis.

Multimedia Appendix 3 Study characteristics, PAEHR functionalities and dimensions of Patient Centered Care.

Multimedia Appendix 4 Analysis of outcomes.

## Abbreviations

PAEHR: patient accessible health records
PAM-13: 13-question Patient Activation Measurement
